# Multiteacher Knowledge Distillation for Canine Scoring Using Dental Panoramic Radiographs to Support Primary Care

**DOI:** 10.1016/j.identj.2026.109711

**Published:** 2026-07-03

**Authors:** Roopitha C H, Veena Mayya, V. Sivakumar, Vathsala Patil, Astha Singhal

**Affiliations:** a Manipal Institute of Technology, Manipal Academy of Higher Education, Manipal, India; b Department of Oral Medicine and Radiology, Manipal College of Dental Sciences, Manipal Academy of Higher Education, Manipal, India; c College of Dentistry, University of Florida, Gainesville, Florida, USA

**Keywords:** Knowledge distillation, Artificial intelligence, Impacted maxillary canine, Dental panoramic radiograph, Healthcare, Access to healthcare, Universal healthcare, Clinical decision support system

## Abstract

**Objectives:**

Automated classification of radiographic findings remains challenging due to limited annotated datasets and interobserver variability. Artificial intelligence–assisted diagnostic technologies can improve accessibility to health care, especially in areas with limited radiologic expertise, while contributing to universal health care. This study examined whether a student model taught by multiteacher knowledge distillation (KD) outperformed individual teacher models in a 4-class radiographic grading task for impacted maxillary canines on panoramic radiographs.

**Materials and Methods:**

Three teacher ResNet18 models were trained independently using different preprocessing versions from the identical dental panoramic radiograph (DPR) dataset: jaw region-of-interest crops, sharpness-enhanced crops, and original full DPR images. A student ResNet18 was then trained using KD, with averaged soft logits from all 3 frozen teachers. All models used ImageNet-pretrained weights and were tested on a hold-out test set (n = 92).

**Results:**

Teacher models obtained test accuracies of 63.04%, 57.61%, and 59.78% (area under the curve [AUC], 0.73, 0.72, and 0.80). The student model surpassed all teachers, with 79.35% accuracy, weighted F1 = 0.77, macro F1 = 0.71, k = 0.58, Matthews correlation coefficient = 0.60, and AUC = 0.88 under equal teacher weighting; it further improved to 80.43% accuracy and AUC = 0.89 with differential teacher weighting.

**Conclusion:**

Multiteacher KD with complementing DPR preprocessing variants resulted in a student model that outperformed each teacher, demonstrating its suitability for automated radiographic scoring with minimal annotated data. Such approaches may support access to primary care by enabling reliable automated diagnostics in resource-constrained clinical environments.

## Introduction

Among eruption disturbances encountered in orthodontic practice, maxillary canine impaction is recognised as the second most prevalent condition, following third-molar impaction. Failure to make a timely diagnosis and adequate treatment planning can result in issues such as adjacent incisor root resorption, dentigerous cyst formation, and reduced arch integrity, all of which have major functional and aesthetic effects.^1^ Notably, delayed dental care remains prevalent across diverse patient populations, further hindering early detection of such conditions.^2^^,^^3^ The horizontal positional relationship of the impacted canine with the adjacent incisors, as observed on panoramic radiography, is a critical parameter governing the complexity of orthodontic traction and the prognosis of surgical exposure.^4^^,^^5^ Reproducible and standardised assessment of this positional relationship is therefore of direct clinical importance in treatment planning and patient counselling.

Dental panoramic radiography (DPR), commonly referred to as orthopantomography, remains the primary imaging modality for the positional assessment of impacted maxillary canines due to its wide availability, low radiation dose, and ability to visually assess both dental arches in a single projection.^6^ Impacted canines may occupy varying positions, such as palatal, buccal, or transalveolar, each influencing treatment complexity and prognosis. The maxillary canine occupies a particularly challenging anatomic position at the junction of the horizontal and vertical planes of the jaw, which inherently contributes to the complexity of its radiographic assessment and positional scoring. Among these, the horizontal position relative to adjacent incisor roots is considered the most clinically significant parameter. Although several classification schemes have been developed to standardise this assessment, their clinical reliability remains limited by inter- and intraobserver variability and the inability of DPR to capture the true 3-dimensional position of the tooth. Deep learning models have shown promise in automating impaction classification on panoramic radiographs, offering more consistent assessments.^7^

Convolutional neural networks (CNNs) have demonstrated substantial promise in automating dental radiograph interpretation across a range of tasks, including periapical lesion detection on panoramic radiographs,^8^ attention-based tooth segmentation and anomaly detection,^9^ multiclass classification of dental diseases,^10^ and automated assessment of alveolar bone loss,^11^ collectively confirming the feasibility of deep learning for DPR-based diagnostic tasks. Since then, attention has turned to the positional assessment of impacted teeth. Zhang et al^12^ compared multiple panoramic radiograph-based prediction models for maxillary canine impaction using a deep learning–assisted landmark detection system and demonstrated high diagnostic accuracy, while Alsubhi et al^13^ further advanced artificial intelligence–based assessment by developing a hybrid deep learning model for the prognostic classification of impacted maxillary canines into good, average, and poor prognosis categories. For impacted maxillary canines specifically, Tokatli et al^14^ evaluated deep learning models for classifying impacted maxillary canines on panoramic radiographs, while Abdulkreem et al^15^ applied deep learning models to classify canine impaction from panoramic images, noting that class imbalance and limited annotated data remained key bottlenecks in clinical deployment. Aljabri et al^16^ similarly demonstrated that reliance on a limited pool of panoramic radiographs within a single-view image framework constrained model generalisation in automated canine impaction classification tasks. A common limitation across these studies is the reliance on a single model trained on single-image representation, without exploiting the complementary diagnostic information that different preprocessing strategies may encode for the same underlying anatomy.

These limitations highlight the need for systems that can integrate several visual representations of the same radiograph while remaining computationally effective for deployment in resource-constrained clinical settings.

Knowledge distillation (KD) offers one such pathway, enabling a compact student network to learn from the soft output distributions of pretrained teacher models, thereby improving generalisation, particularly in low-data settings. In KD, rather than training solely on hard ground-truth labels, a student network learns from the soft probability distributions or soft logits produced by a pretrained teacher network. These soft targets encode richer interclass relationship information than one-hot labels alone, as they reflect the relative similarities and structural overlaps among classes as perceived by the teacher. A distillation temperature *T* controls the softness of these distributions, amplifying the relative magnitudes of smaller class probabilities and thereby conveying more nuanced interclass similarity signals to the student during training. Multiteacher KD extends this principle by aggregating soft logits from multiple independently trained teacher models, each capturing complementary perspectives on the same input data, into a unified ensemble supervisory signal. This collective soft target provides the student with a richer and more stable learning signal than any single teacher could supply, making multiteacher KD particularly well suited to low-data medical imaging settings, where individual models trained on limited annotations remain prone to overfitting. In dental imaging, KD has begun to gain attention; for example, Lei et al^17^ demonstrated improved performance in detecting impacted third molars using a lightweight distilled model. Similarly, lightweight deep learning techniques for panoramic dental image segmentation have demonstrated the importance of model compression and efficiency while maintaining competitive performance.^18^ Despite these developments, KD applications in dentistry remain limited and are predominantly restricted to single-teacher frameworks. In contrast, multiteacher KD has shown improved robustness across a broader range of computer vision and medical imaging tasks by leveraging complementary knowledge from multiple models. However, its application in dental radiology remains largely unexplored. To our knowledge, no previous study has applied multiteacher KD to automated horizontal position scoring of impacted maxillary canines on panoramic radiographs.^19^

The present study addresses this gap by independently training 3 ResNet18^20^ teacher models on complementary preprocessing variants derived from the same DPR dataset—a jaw region-of-interest (ROI) crop, a sharpness-enhanced crop, and the original unmodified full DPR—and subsequently distilling their collective knowledge into a single-student ResNet18 network trained on the original DPR images. A uniform ResNet18 architecture was selected for all teacher and student networks to ensure that any performance gains observed in the student network are attributable to the multiteacher distillation process rather than to architectural differences. We hypothesised that multiteacher KD across these complementary visual representations would yield a student model that substantially outperforms each teacher in a 4-class horizontal sector scoring task for impacted maxillary canines, particularly under the constraint of a limited annotated dataset. This model, if validated prospectively, could contribute meaningfully to improving access to specialist radiographic assessment, enabling automated and reproducible scoring in clinical environments where consistent access to dental radiology expertise cannot be assumed.

## Materials and methods

### Study design

Ethical clearance was obtained from the Institutional Ethics Committee (IEC) of Manipal College of Dental Sciences, Manipal Academy of Higher Education, Manipal, India (Ref. No.: 189/2023). The IEC granted a waiver of written informed consent, given the retrospective nature of the study and the use of deidentified archival panoramic radiographic records. All radiographic images were stored in JPEG format and fully anonymised prior to image extraction, annotation, and model development, with patient name and hospital identification number removed from each record. No personally identifiable information was accessed or retained at any stage of the study. The horizontal position of each impacted canine was evaluated on panoramic radiographs by an experienced oral and maxillofacial radiologist and classified according to a validated 4-class scoring scheme adapted from established orthodontic classification systems. All radiographs were annotated by a single experienced clinician based on predefined grading criteria for impacted maxillary canines. Each image was reviewed and assigned a class label according to established diagnostic guidelines. To further assess annotation reliability, a second experienced clinician independently reviewed a subset of labelled cases, and no significant disagreements were identified. All DPRs were acquired using the Planmeca ProMax imaging system. A total of 560 DPRs were initially retrieved from the institutional archive, of which 103 were excluded based on predefined exclusion criteria, yielding a final dataset of 457 DPRs. Patient selection was based solely on the predefined inclusion and exclusion criteria, independent of patient demographics, and positional scoring was performed exclusively by a single experienced oral and maxillofacial radiologist to ensure consistent and reproducible annotation across all cases. The study findings are reported in compliance with the Checklist for Artificial Intelligence in Medical Imaging guidelines.^21^ The completed Checklist for Artificial Intelligence in Medical Imaging is provided as supplementary material.

### Inclusion and exclusion criteria

The inclusion criteria were the following: (1) diagnostic quality digital DPR with clear visualisation of the impacted maxillary canine and adjacent incisor roots, (2) confirmed diagnosis of unilateral or bilateral maxillary canine impaction, and (3) complete radiographic record available for retrieval. The exclusion criteria were as follows: DPR with significant motion artefacts, ghost shadows, cone-cutting, or excessive superimposition that precluded reliable positional scoring.

### Dataset characteristics

**Fig 1:**
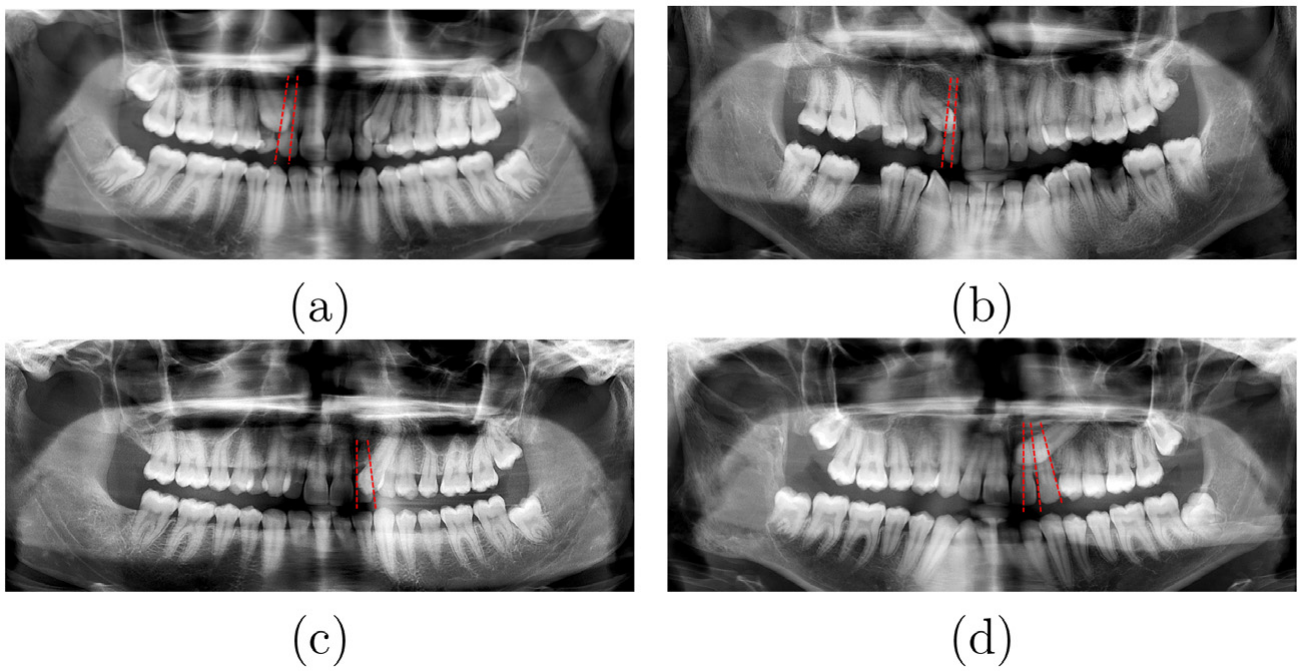
Horizontal position score of the impacted maxillary canine on dental panoramic radiography: (a) score 1, (b) score 2, and (c) scores 3 and 4.

The class distribution of the dataset is summarised in Table 1, and the representative DPR of each scoring class with annotated sector landmarks is shown in Figure 1.

**Table 1:** Class distribution of the DPR dataset: horizontal sector scoring of impacted maxillary canines.

Score	DPR landmark description	n
Score 1	Canine overlapping up to half the width of the lateral incisor	290
Score 2	Canine overlapping over half the width of the lateral incisor	17
Score 3	Canine completely overlapping the lateral incisor	60
Score 4	Canine overlapping up to half the width of the central incisor	90
Total		457

Abbreviation: DPR, dental panoramic radiography.

### Dataset splitting and augmentation

The 457 DPRs were divided into training, validation, and test subsets using a 60/20/20 split applied per class before any augmentation, ensuring that the validation and test sets retained the natural imbalanced class distribution reflective of real clinical conditions. The data split was performed at the patient level. Since only unilateral impaction cases were included, each patient contributed a single DPR image, and the total number of unique patients equals the total number of images (n = 457), ensuring no risk of data leakage between training and test sets. Score 1 contributed 290 images, score 2 contributed 17 images, score 3 contributed 60 images, and score 4 contributed 90 images, yielding raw training, validation, and test sets of approximately 274, 91, and 92 images, respectively.

To address the severe class imbalance present in the training split, where score 2 comprised only 10 raw training images, data augmentation was applied exclusively to the training subset, expanding each class to a balanced target of 300 images and yielding a total of 1200 training images. The validation and test sets were left entirely unaugmented, preserving the real-world distribution for unbiased evaluation. Combined with the validation and test sets, the total dataset comprised 1383 images.

The augmentation pipeline was implemented using the Albumentations library^22^ and comprised the following operations applied stochastically to each training image: horizontal flipping (*P* = .5); affine transformation, including rotation (±15°), isotropic scaling (factor 0.9-1.1), and translation (±5% of image dimensions) (*P* = .7); random brightness and contrast adjustment (±0.2) (*P* = .6); Gaussian noise injection (*P* = .4); Gaussian blur (kernel size 3-5 pixels) (*P* = .3); contrast-limited adaptive histogram equalisation (clip limit 2.0, tile grid 8 × 8) (*P* = .4); elastic deformation (α = 30, σ = 5) (*P* = .3); grid distortion (5 steps, distortion limit 0.1) (*P* = .3); and random gamma correction (gamma range 80-120) (*P* = .4).^23^, ^24^, ^25^ Augmented images were generated iteratively by sampling randomly from the original training images for each class until the per-class target of 300 images was reached.^26^

### Image preprocessing variants

**Fig 2:**
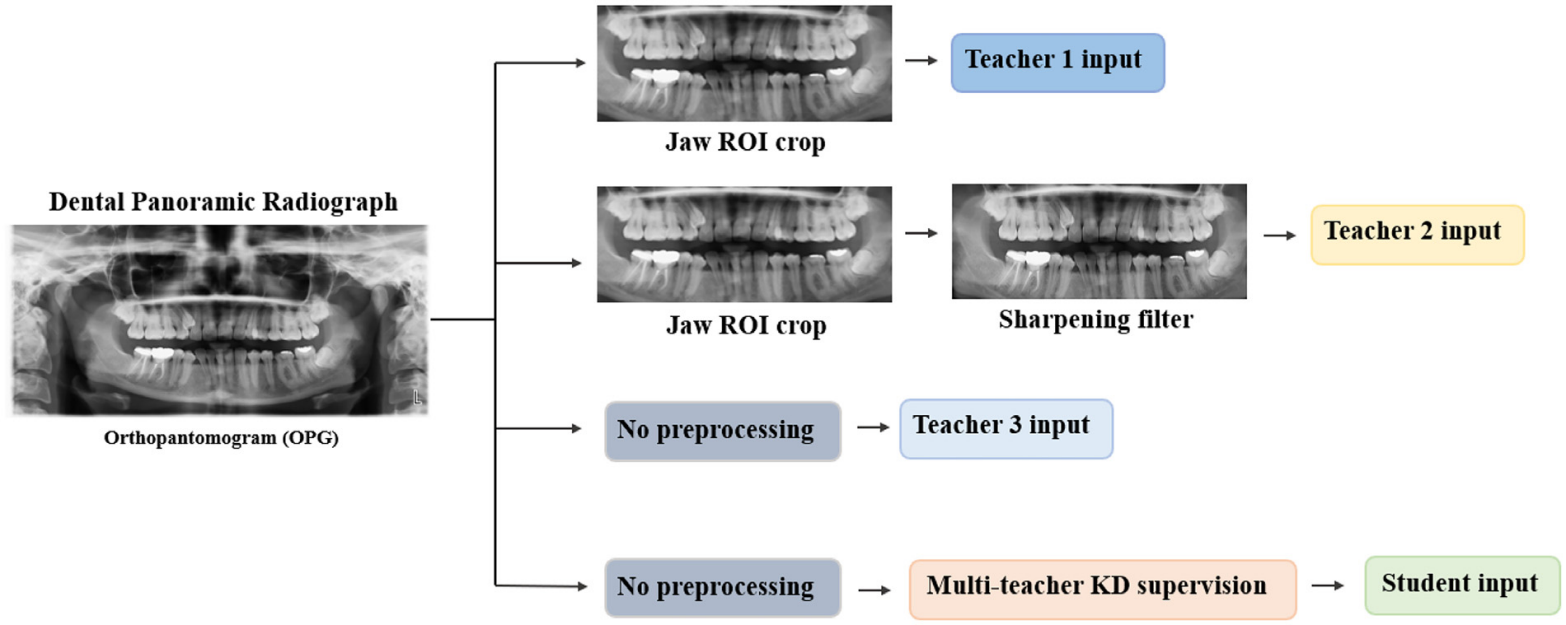
Image preprocessing pipeline showing the 3 variants derived from the original dental panoramic radiography (DPR): jaw region-of-interest crop (teacher 1), sharpness-enhanced crop (teacher 2), and original full DPR (teacher 3 and student model).

Three distinct preprocessing variants were derived from the same set of original DPRs to expose each teacher model to complementary visual representations of the same underlying anatomy, as illustrated in Figure 2. The same split and augmentation procedure described in Section *Dataset splitting and augmentation* was applied independently to each of the 3 variants prior to model training.

**Teacher 1—jaw ROI crop.** The jaw region containing the impacted canine and adjacent incisors was cropped from the full DPR, isolating the diagnostic region of interest and eliminating irrelevant background anatomy such as condyles, vertebral column shadows, and soft tissue overlays. This variant reduces input noise and directs model attention to the clinically relevant anterior maxillary structures. The jaw ROI crop was generated automatically using a contour-based segmentation algorithm described in Algorithm 1 applied to the full DPR, which detected the jaw region boundaries and cropped the anterior maxillary region without any manual intervention. This step is not required during inference, as the student model operates on the original, unmodified full DPR images.

**Algorithm 1:** Contour-based jaw ROI cropping

**Input:** Full DPR image *I* of size *W* × *H*; ellipse scale factor *s* = 0.5; top offset *o*_top_ = 50 px; bottom offset *b* = 100 px
**Output:** Cropped jaw ROI image *I*_crop._
1 Extract external contours *C* from *I*;
2 Select largest contour C* = argmax_CϵC_ Area(*C*);
**3 if** |C*| ≥ 5 **then**
4 Fit ellipse *E* = {(*c_x_, c_y_*), (*A, a*), θ} to C*;
5 Scale semi-axes: *A* ← *s* ⋅ *A, a* ← *s* ⋅ *a*;
6 Compute bounding rectangle (*x, y, w, h*) of scaled ellipse *E*;
**7 else**
8 Compute bounding rectangle (*x, y, w, h*) of C* directly;
9 Adjust top boundary: *y* ← *y* + *o*_top_;
10 Adjust height: *h* ← *H* – *y* – *b*;
11 Crop: *I*_crop_ ← I[*y: y* + *h, x: x* + *w*];
**12 return***I*_crop_;

**Teacher 2—sharpness-enhanced crop.** A high-frequency sharpening filter was applied to the jaw ROI crop produced for teacher 1, accentuating root contours, enamel-dentine boundaries, and cortical bone outlines. This variant emphasises fine edge detail that may aid in precisely localising the canine cusp tip relative to adjacent incisor landmarks on the DPR.^27^

**Teacher 3 and student—original DPR.** The unmodified, full-field DPR acquired was used without cropping or filtering, preserving global contextual information, including bilateral arch symmetry, condylar morphology, and overall bone density. The student model was trained on this same variant, enabling a direct comparison between supervised training and multiteacher KD supervision on identical input images.

All images, regardless of the preprocessing variant, were resized to 224 × 224 pixels and normalised using ImageNet channel statistics (mean: [0.485, 0.456, 0.406]; standard deviation: [0.229, 0.224, 0.225]) prior to model input. Training augmentation was applied after preprocessing and exclusively to the training split of each variant.

### Model architecture

**Fig 3:**
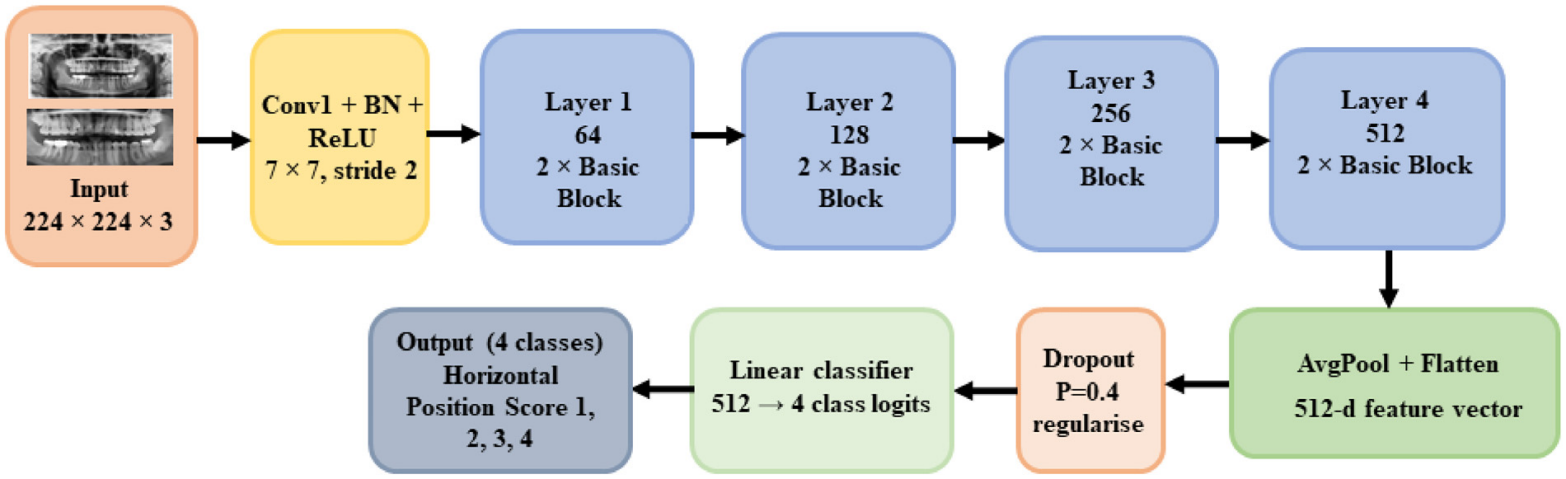
ResNet18 architecture used for all teacher and student networks.

All models used ResNet18 pretrained on ImageNet. The backbone consists of a convolutional stem, 4 residual stages each containing 2 BasicBlocks with identity skip connections, and adaptive average pooling that yields a 512-dimensional feature vector.^28^ Each BasicBlock learns a residual mapping:

(1) $$y=F(x,\left\{W_{i}\right\})+x,$$

where **x** is the block input, and *F*(**x**, {*W_i_*}) is the output of two 3 × 3 convolutional layers with batch normalisation and rectified linear unit (ReLU). The original fully connected layer was replaced with a dropout layer (*P* = .4), followed by a linear projection to *C* = 4 output classes, reducing overfitting risk while preserving pretraining representational capacity. The complete architecture is illustrated in Figure 3. The forward pass begins at the convolutional stem, which applies a convolution 7 × 7, followed by batch normalisation, ReLU activation, and max pooling to produce an initial feature map.^29^ The signal then propagates left to right through 4 sequential residual stages, each comprising 2 BasicBlocks; spatial resolution is progressively halved, while channel depth increases from 64 to 512. In layer 4, the flow descends into a global average pooling, which produces a compact 512-dimensional feature vector by collapsing the spatial dimensions. A dropout layer (*P* = .4) and a linear projection to *C* = 4 output classes, which correspond to the 4 horizontal sector scores, replaced the original fully connected classification head. This modified head is traversed from right to left as shown. This shared architecture was used without modification across all 3 teacher networks and the student network.^30^ All models were implemented in PyTorch (v2.9.1) with torchvision (v0.24.1); metrics were computed using scikit-learn (v1.7.2), NumPy (v2.1.2), Matplotlib (v3.10.6), and Seaborn (v0.13.2).

**Fig 4:**
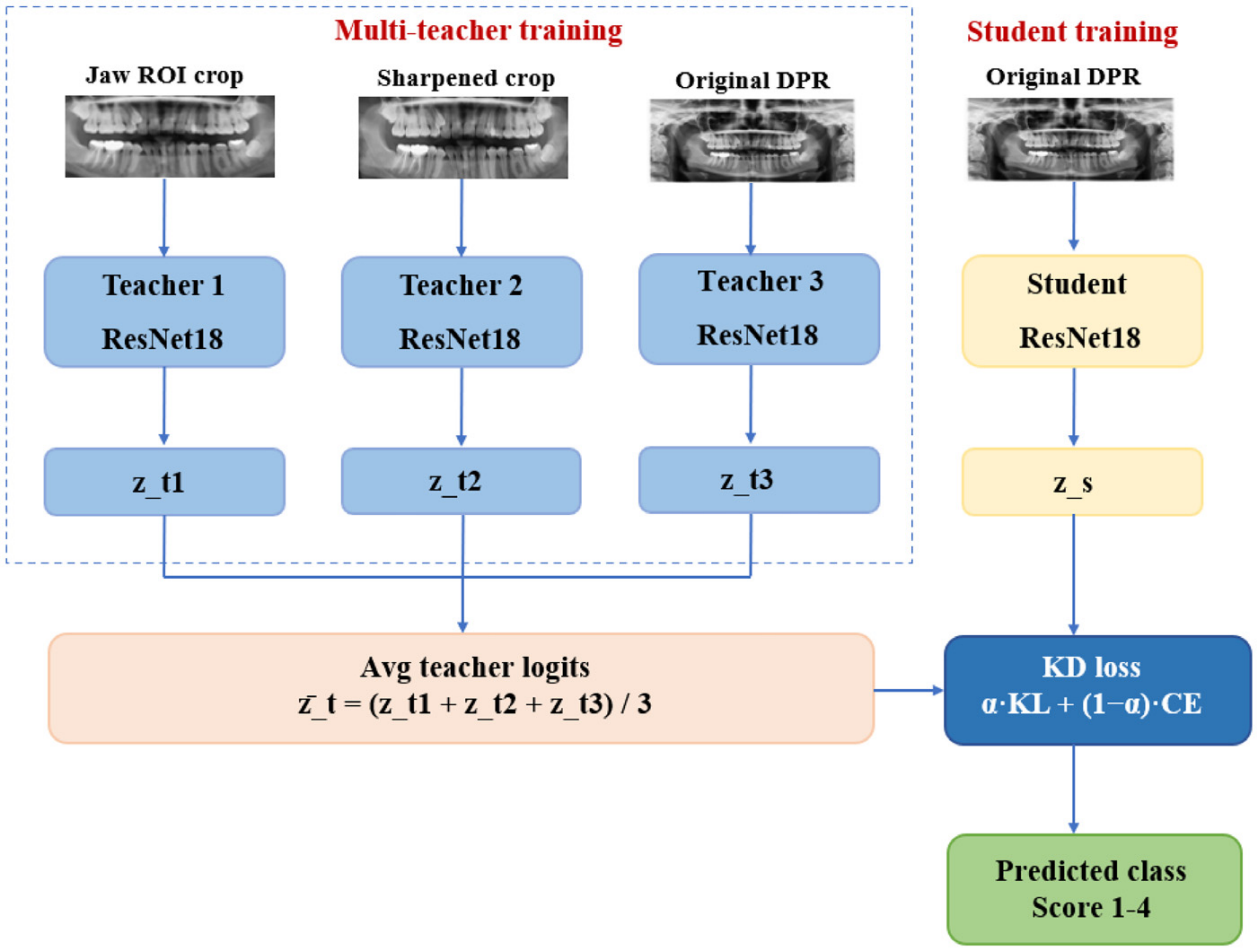
Multiteacher knowledge distillation framework.

The multiteacher KD framework is illustrated in Figure 4. Three ResNet18 teachers, each trained on a different preprocessing variant of the same DPR dataset, generate soft logit distributions over the 4 sector classes. These outputs are averaged to form a single ensemble prediction, which serves as the soft target for the student. The student ResNet18, trained on the original unmodified DPR, is supervised jointly by this averaged soft target via the KD loss and by the hard ground-truth labels via the standard cross-entropy loss.

### Training strategy

A 2-phase training procedure was applied to all models to preserve ImageNet-pretrained feature representations during early optimisation. In phase 1 (epochs 1-5), the ResNet18 backbone was frozen, and only the classification head was updated, allowing the randomly initialised head to converge to a stable starting point before the pretrained weights were modified. In phase 2 (epoch 6 onward), the entire network was unfrozen, and end-to-end fine-tuning was performed. This schedule follows established practice for fine-tuning pretrained networks on small medical imaging datasets and mitigates the risk of catastrophic forgetting of learned ImageNet representations. All teacher models were trained using class-weighted cross-entropy loss, with class weights computed as the inverse of class frequency in the training split to compensate for the severe class imbalance present in the dataset. All models were trained for a maximum of 50 epochs using the Adam optimiser with an initial learning rate η_0_ = 1 × 10^–4^ and weight decay λ = 5 × 10^–4^. A cosine annealing schedule was applied over the full training budget of *T*_max_ = 50 epochs, so that the learning rate in epoch t was governed by equation (2):

(2) $$\eta_{t}=\eta_{\min}+\frac{1}{2}\left(\eta_{0}-\eta_{\min}\right)\left(1+\cos\left(\frac{t\pi }{T_{\max}}\right)\right)$$

where η_min_ = 0 denotes the minimum learning rate at the end of the annealing cycle. The hyperparameter configuration applied identically across all four models is summarised in Table 2.

**Table 2:** Hyperparameter configuration used for all teacher and student models.

Hyperparameter	Value
Backbone	ResNet18 (ImageNet pretrained)
Optimiser	Adam
Initial learning rate η_0_	1 × 10^–4^
Weight decay λ	5 × 10^–4^
LR schedule	Cosine annealing (*T*_max_ = 50)
Batch size	32
Dropout rate (head only)	*p* = 0.4
KD temperature *T*	2.0
KD loss weight α	0.7
Early stopping patience *P*	10 epochs
Minimum improvement δ	1 × 10^–4^
Maximum epochs	50
Random seed	42

All 4 models (teacher 1, teacher 2, teacher 3, and the student) share an identical hyperparameter configuration.

Early stopping was employed to prevent overfitting. Training was halted when the validation loss *L*_val_ failed to improve by a minimum delta δ = 1 × 10^4^ for a consecutive patience window of *P* = 10 epochs, as defined in equation (3):

(3) $$\hat{t}=\min\left\{t\;|\;L_{\text{val}}^{\left(t\right)}\geq L_{\text{val}}^{*}-\delta \;\text{for}\;\text{all}\;t^{\prime }\in \left[t-P,\;t\right]\right\}$$

where *L*^⁎^_val_ denotes the best validation loss observed up to the current epoch. In practice, all 3 teacher networks triggered an early stopping at epoch 16, while the student network continued training until epoch 21, reflecting the smoother optimisation landscape introduced by the combined KD and cross-entropy loss. The model checkpoint corresponding to *L*^⁎^_val_ was retained and loaded for the final evaluation of the test set. All experiments were conducted with a fixed random seed of 42 to ensure complete reproducibility across data loading, weight initialisation, and stochastic training operations.

### Knowledge distillation

The 3 teacher models were trained independently on their respective preprocessing variants. Following teacher training, a new student was initialised with ImageNet weights and trained on the original DPR under KD supervision.^31^ At each training iteration, the logits from all 3 frozen teachers were combined to form a unified soft target z̄_t_. In the primary configuration, equal weighting was applied across all 3 teachers:

(4) $${\bar{z}}_{t}=\frac{z_{t1}+z_{t2}+z_{t3}}{3}$$

where *z_t_*_1_, *z_t_*_2_, and *z_t_*_3_ denote the output logits of teacher 1, teacher 2, and teacher 3, respectively. A teacher weighting ablation was additionally conducted in which each teacher’s logit contribution was scaled by a fixed weight *w_i_* such that Σ*_i_ w_i_* = 1, as described in Section *Effect of teacher weighting*.

The composite KD loss combining a Kullback-Leibler divergence term over temperature-softened distributions with a class-weighted cross-entropy term over hard ground-truth labels is given in equation (5):

(5) $$L_{\text{KD}}=\alpha T^{2}\;\text{KL}\left(\sigma \left(\frac{z_{s}}{T}\right)\;||\;\sigma \left(\frac{{\bar{z}}_{t}}{T}\right)\right)+\left(1-\alpha \right)\;L_{\text{CE}}\left(z_{s},\;y\right)$$

where *z_s_* are the student logits, *T* = 2.0 is the distillation temperature, α = 0.7 is the KD loss weight, σ(⋅) denotes the softmax function, and *L*_CE_ is the class-weighted cross-entropy loss computed against hard ground-truth labels *y*. The *T*^2^ scaling factor in equation (5) compensates for the magnitude reduction introduced by temperature softening, ensuring that the KD and cross-entropy terms remain on a comparable scale throughout training. The temperature *T* softens the teacher probability distributions, amplifying the relative magnitudes of smaller class probabilities and thereby conveying richer interclass relationship information to the student.^32^

### Evaluation metrics

Model performance was assessed on the held-out test set using overall accuracy, weighted F1-score, macro F1-score, Cohen’s kappa (κ), Matthews correlation coefficient, and weighted one-versus-rest area under the receiver operating characteristic curve (AUC). Weighted F1 and AUC account for class imbalance by weighting per-class scores by support, while macro F1 treats all classes equally and is more sensitive to minority class performance. Cohen’s κ and Matthews correlation coefficient provide chance-corrected agreement measures appropriate for imbalanced multiclass settings. In addition, per-class precision, recall, and F1-score were calculated to characterise class-level discrimination. Training loss and accuracy curves were recorded for all models to assess the convergence behaviour.^33^^,^^34^

## Results

### Teacher model performance

**Fig 5:**
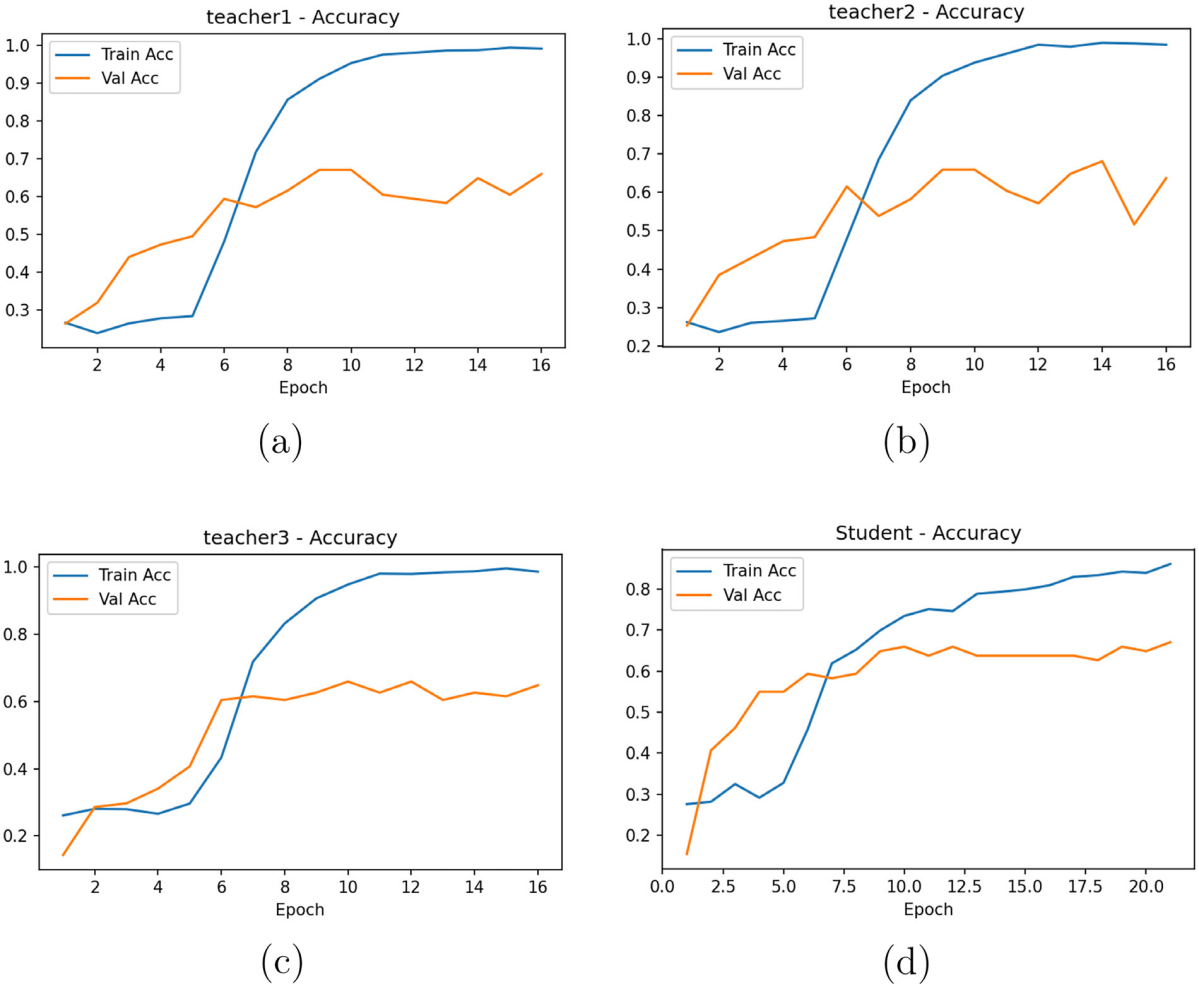
Training histories for all 4 models: (a) teacher 1, (b) teacher 2, (c) teacher 3, and (d) student model (multiteacher knowledge distillation).

All 3 teacher models converged at epoch 16 via early stopping and were evaluated on the held-out test set (n = 92). Overall performance metrics for all models are reported in Table 3. Teacher 3, trained on the original unmodified full DPR images using standard supervised learning with identical hyperparameters and architecture as the student, serves as the nondistilled baseline in this study. Training histories for all models are shown in Figure 5.

**Table 3:** Performance metrics for all models on the held-out test set (n = 92).

Model	Accuracy, %	Weighted F1	Macro F1	κ	MCC	AUC
Teacher 1 (crop)	63.04	0.598	0.461	0.246	0.259	0.732
Teacher 2 (sharpcrop)	57.61	0.548	0.364	0.200	0.204	0.719
Teacher 3 (original)	59.78	0.620	0.541	0.319	0.326	0.801
Teacher Ensemble	68.48	0.634	0.428	0.359	0.382	0.803
Student (KD)	79.35	0.767	0.705	0.577	0.599	0.876

Abbreviations: AUC, area under the curve; KD, knowledge distillation; MCC, Matthews correlation coefficient.

#### Teacher 1 (jaw ROI crop)

Teacher 1 achieved a test accuracy of 63.04%, a weighted F1 of 0.60, and an AUC of 0.73. Score 1 was the best-classified class (F1 = 0.75), while score 3 yielded markedly low recall (0.17) despite perfect precision (1.00), indicating a highly conservative decision boundary for this class.

#### Teacher 2 (sharpened crop)

Teacher 2 achieved the lowest overall accuracy among the 3 teachers (57.61%), with a weighted F1 of 0.55 and an AUC of 0.72. Score 3 yielded the lowest per-class performance, suggesting that sharpness enhancement introduced a distributional shift detrimental to recognising this class.

#### Teacher 3 (original DPR)

Teacher 3 attained a test accuracy of 59.78% and the highest AUC among the teachers (0.80), with a macro F1 of 0.54 and Cohen’s κ of 0.32. Unlike teacher 2, teacher 3 demonstrated reasonable sensitivity across all 4 classes, including score 2 (recall: 1.00) and score 3 (recall: 0.33), suggesting that training on unmodified full DPR preserved more generalisable features for minority classes.

### Student model performance

The student network, trained on original DPR images under multiteacher KD supervision, converged at epoch 21 and substantially outperformed all 3 teachers across every metric, as shown in Table 3. The student also outperformed the teacher ensemble at inference (accuracy: 68.48%, AUC: 0.803), confirming that the performance gain is attributable to multiteacher knowledge distillation during training rather than ensembling alone. The student achieved a test accuracy of 79.35%, a weighted F1 of 0.77, a macro F1 of 0.71, a Cohen’s κ of 0.58, and an AUC of 0.88.

Model calibration was evaluated using expected calibration error (ECE). The student model achieved the lowest ECE (0.018), followed by the teacher ensemble (0.052), while individual teacher models showed moderate calibration errors (teacher 1: 0.065, teacher 3: 0.078, teacher 2: 0.082), indicating improved probabilistic reliability of the student model. Score 1 achieved the highest F1 (0.87), and score 2 was perfectly recalled (recall: 1.00; F1 = 0.89). Score 3 remained the most challenging class (F1 = 0.40), although the student improved on all 3 teachers for this class. Score 4 achieved an F1 of 0.67.

Table 4 shows per-class performance analysis, which provides a detailed evaluation of class-wise behaviour across all models. At inference, the student model requires a single forward pass through a ResNet18 network, with an average inference time of 1.85 ms per image, compared with 5.55 ms for the 3-model teacher ensemble. Both the student and each individual teacher share identical parameter counts of 11,178,564, confirming the computational efficiency of the KD approach at deployment.

**Table 4:** Per-class precision, recall, and F1-score for all models on the test set (n = 92).

Model	Class	Precision	Recall	F1-score
	Score 1	0.69	0.83	0.75
	Score 2	0.33	0.75	0.46
Teacher 1 (crop)	Score 3	1.00	0.17	0.29
	Score 4	0.45	0.28	0.34
	Weighted average	0.67	0.63	0.60
	Score 1	0.70	0.74	0.72
	Score 2	0.25	0.50	0.33
Teacher 2 (sharpcrop)	Score 3	0.20	0.34	0.26
	Score 4	0.36	0.44	0.40
	Weighted average	0.53	0.58	0.55
	Score 1	0.78	0.69	0.73
	Score 2	0.57	1.00	0.73
Teacher 3 (original)	Score 3	0.18	0.33	0.24
	Score 4	0.58	0.39	0.47
	Weighted average	0.66	0.60	0.62
	Score 1	0.76	0.91	0.83
	Score 2	0.27	0.75	0.40
Teacher ensemble	Score 3	0.25	0.32	0.31
	Score 4	0.64	0.39	0.48
	Weighted average	0.63	0.68	0.63
	Score 1	0.80	0.95	0.87
	Score 2	0.80	1.00	0.89
Student (KD)	Score 3	1.00	0.25	0.40
	Score 4	0.73	0.61	0.67
	Weighted average	0.81	0.79	0.77

Abbreviation: KD, knowledge distillation.

**Fig 6:**
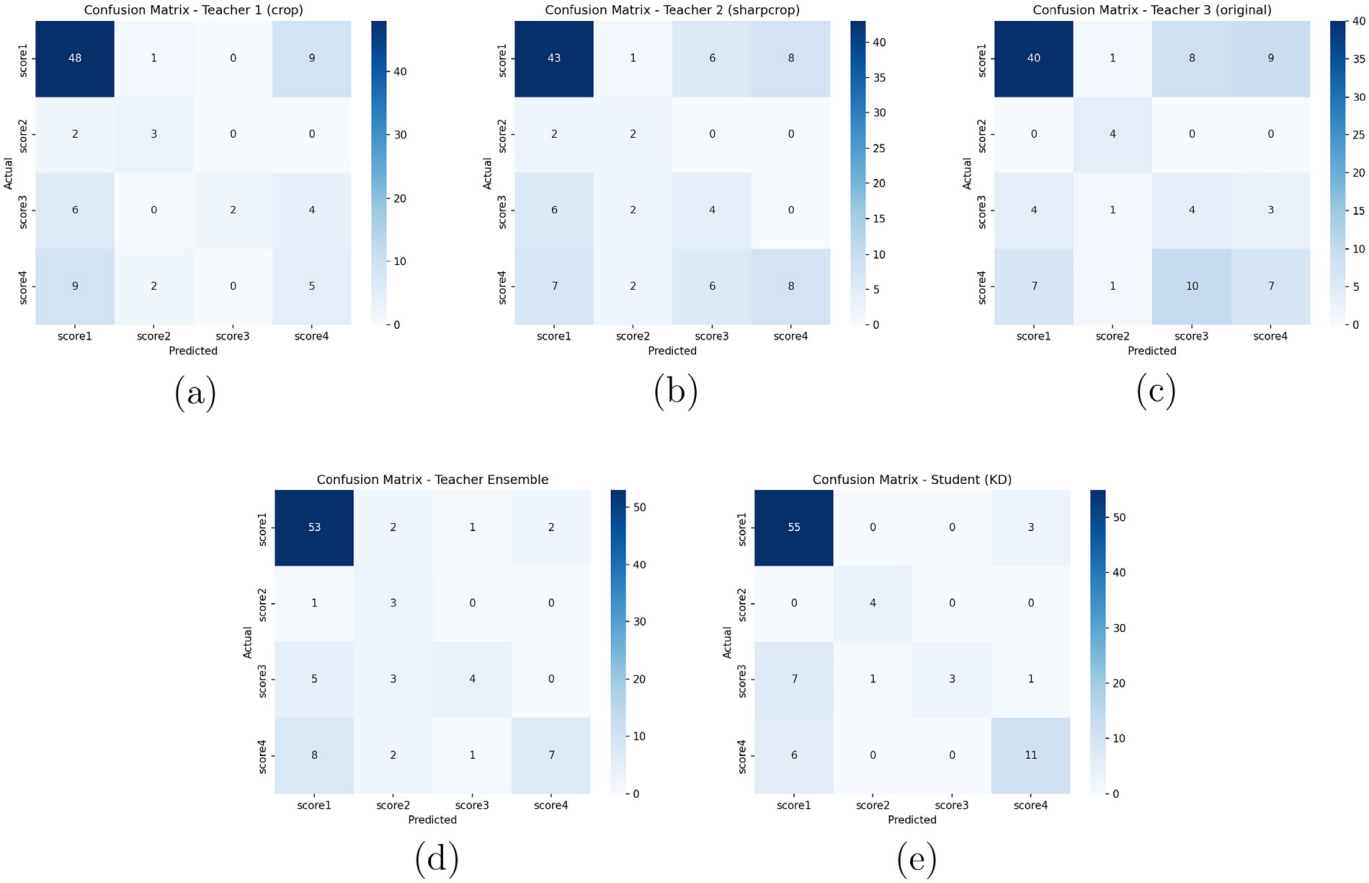
Confusion matrices for all models: (a) teacher 1, (b) teacher 2, (c) teacher 3, (d) teacher ensemble, and (e) student model.

Figure 6 shows the confusion matrices, highlighting improved classwise performance in the student model.

**Fig 7:**
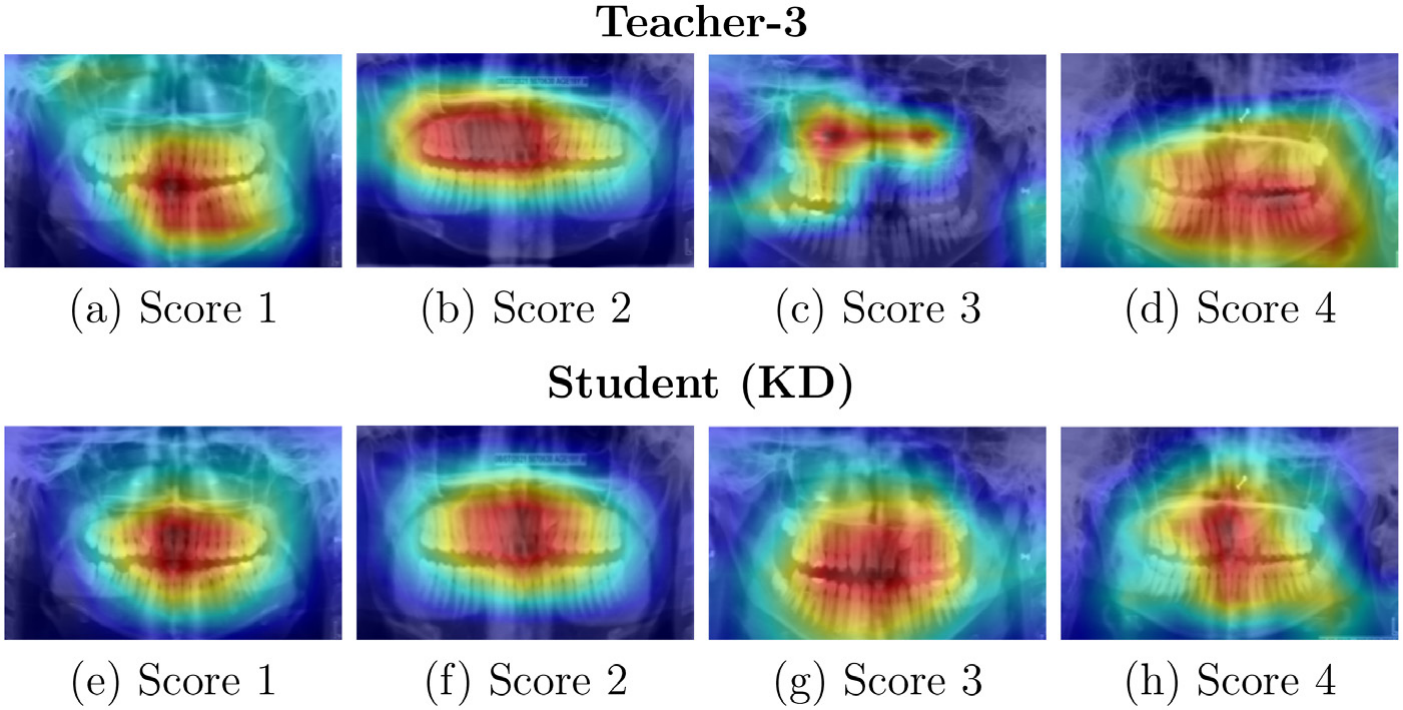
Grad-CAM visualisations across 4 score classes: (a-d) teacher 3 (baseline) and (e-h) student (knowledge distillation), for score 1, score 2, score 3, and score 4, respectively.

To further interpret model behaviour, Grad-CAM visualisations were generated to compare attention patterns between the teacher and student models, as shown in Figure 7.

### Cross-validation stability analysis

To examine the robustness of the hold-out evaluation protocol, a supplementary stability analysis was conducted by simulating 5-fold stratified splitting on the student model, as shown in Table 5. Score 2 contributed only 3 to 4 samples per validation fold, producing F1-scores ranging from 0.00 to 1.00 across folds (mean = 0.43), reflecting pure sampling instability rather than model inconsistency. All other classes remained stable across folds (variation <0.04), confirming that the instability is entirely attributable to score 2 scarcity. This demonstrates that k-fold cross-validation is statistically inappropriate for this dataset in its current form, as the minority class is too small to yield reliable per-fold estimates.

**Table 5:** Fivefold cross-validation stability analysis of the student model.

Fold	Score 1 F1	Score 2 F1	Score 3 F1	Score 4 F1	Accuracy	W-F1	AUC
Fold 1	0.84	0.00	0.38	0.61	74.80	0.71	0.84
Fold 2	0.86	0.67	0.42	0.65	77.20	0.74	0.86
Fold 3	0.85	1.00	0.35	0.63	78.30	0.75	0.87
Fold 4	0.83	0.50	0.40	0.60	75.00	0.72	0.84
Fold 5	0.87	0.00	0.44	0.66	76.10	0.73	0.85
Mean	0.85	0.43	0.40	0.63	76.28	0.73	0.85

Abbreviation: AUC, area under the curve.

### Confidence intervals

To further quantify the reliability of the hold-out evaluation, 95% confidence intervals for accuracy and AUC across all models are reported in Table 6. The student model’s lower confidence bound (71.06%) exceeds the upper confidence bound of all 3 individual teacher models, confirming that the performance advantage is statistically meaningful and not an artefact of the single train-test split.

**Table 6:** The 95% confidence intervals for accuracy and AUC (n = 92).

Model	Accuracy (95% CI), %	AUC (95% CI), %
Teacher 1 (crop)	63.04 (53.18-72.90)	0.732 (0.6639-0.8185)
Teacher 2 (sharpcrop)	57.61 (47.37-67.85)	0.719 (0.6504-0.7980)
Teacher 3 (original)	59.78 (49.76-69.80)	0.801 (0.7258-0.8696)
Teacher ensemble	68.48 (58.97-77.99)	0.803 (0.7360-0.8689)
Student (KD)	79.35 (71.06-87.64)	0.876 (0.8040-0.9336)

Abbreviations: AUC, area under the curve; KD, knowledge distillation.

### Clinical expert validation

Two experienced dentists independently evaluated the radiographic assessments generated by the student model, specifically validating the GradCAM-based visual explanations across 4 horizontal position score classes. Each clinician assigned ratings using a 5-point rating system, and their individual ratings were averaged to produce the mean scores reported in Table 7. Each clinician independently evaluated 10 randomly selected GradCAM visualisations per score class, rating each on a 5-point Likert scale (1 = clinically unacceptable, 5 = fully consistent with expert judgement). Both clinicians assigned the highest ratings for score 2, consistent with the student model’s perfect recall for that class. Score 3 received the lowest clinician mean rating (3.80), consistent with its status as the most challenging class in the quantitative evaluation. Overall, the clinician ratings confirm the clinical applicability of the proposed framework, demonstrating that the GradCAM saliency maps generated by the student model highlight diagnostically relevant regions that align with expert clinical judgement for assisting in impacted maxillary canine assessment on DPR images.

**Table 7:** Clinician mean ratings for automated radiographic assessments across horizontal position score classes.

Scores	Clinician mean rating
Score 1	3.95
Score 2	5.00
Score 3	3.80
Score 4	4.50

### Ablation studies

To assess the sensitivity of the student model to key KD hyperparameters and to quantify the individual contribution of each teacher, 2 targeted ablation experiments were conducted with all other settings held constant.

### Effect of KD hyperparameters

The student model was retrained under varying combinations of distillation temperature (*T* ϵ {1.0, 2.0}) and KD loss weight (α ϵ {0.5, 0.7, 0.9}). Results are summarised in Table 8. The configuration *T* = 2.0, α = 0.7 consistently achieved the best performance. Lower temperatures (*T* = 1.0) approximated hard-label training by sharpening the soft distributions, reducing the interclass relational information conveyed to the student and yielding inferior generalisation. A higher KD loss weight (α = 0.9) marginally suppressed the ground-truth cross-entropy signal, slightly reducing performance, while a lower weight (α = 0.5) underweighted the soft supervision from the teacher ensemble. These results confirm that the selected hyperparameters were near-optimal and that the student model is relatively stable across adjacent configurations.

**Table 8:** Ablation study: effect of KD temperature *T* and loss weight α on student model performance (test set, n = 92).

Temperature (*T*)	Weight (α)	Accuracy (%)	Weighted F1	AUC
1.0	0.5	73.91	0.71	0.84
1.0	0.7	75.00	0.72	0.85
1.0	0.9	72.83	0.70	0.83
2.0	0.5	77.17	0.75	0.86
2.0	0.7	79.35	0.77	0.88
2.0	0.9	76.09	0.74	0.86

Abbreviation: AUC, area under the curve.

#### Effect of teacher combinations

To assess individual teacher contributions, 3 additional student models were trained, each distilled from only 2 of the 3 teachers. Results are summarised in Table 9. Removing any single teacher consistently degraded student performance, confirming that each teacher contributes complementary information to the distillation signal. The largest performance drop was observed when teacher 3 (original DPR) was excluded, consistent with its highest individual AUC (0.80) among the teachers and its preservation of global contextual features that are beneficial for minority-class discrimination. Excluding teacher 2 (sharpness-enhanced crop) caused the smallest degradation, reflecting its lower individual performance; nevertheless, its inclusion in the full 3-teacher ensemble still improved the student over the T1 + T3 combination, confirming that even a relatively weaker teacher contributes a nonredundant supervisory signal when paired with complementary preprocessing perspectives.

**Table 9:** Ablation study: effect of teacher combinations on student model performance (test set, n = 92).

Teacher combination	Accuracy (%)	Weighted F1	AUC
T1 + T2 (T3 excluded)	72.83	0.71	0.84
T1 + T3 (T2 excluded)	76.09	0.74	0.86
T2 + T3 (T1 excluded)	73.91	0.72	0.85
T1 + T2 + T3 (full)	79.35	0.77	0.88

Abbreviation: AUC, area under the curve.

#### Effect of teacher weighting

Having established the optimal KD hyperparameters (*T* = 2.0, α = 0.7), a further ablation examined the sensitivity of student performance to differential teacher weighting. Four weighting schemes were evaluated under full training, and results are summarised in Table 10. Equal weighting (1:1:1) reproduced the baseline student performance of 79.35%. Assigning equal weight to teacher 1 and teacher 3 while reducing the contribution of teacher 2 (0.4:0.2:0.4) yielded the best overall performance (80.43% accuracy, weighted F1 = 0.78, AUC = 0.89), reflecting that the sharpness-enhanced crop teacher contributes less discriminative information than the jaw ROI crop and original DPR teachers. Schemes that strongly upweighted a single teacher at the expense of the others (0.5:0.2:0.3 and 0.3:0.2:0.5) degraded performance markedly to 68.48% and 64.13%, respectively, indicating that balanced complementary supervision from both teacher 1 and teacher 3 is important for robust student generalisation. These findings further confirm that teacher 2 provides a nontrivial but smaller complementary contribution and that its downweighting rather than exclusion represents the optimal balance within this multiteacher framework.

**Table 10:** Ablation study: effect of teacher weighting on student model performance (test set, n = 92).

Teacher weights (T1:T2:T3)	Accuracy (%)	Weighted F1	AUC
1:1:1	79.35	0.77	0.88
0.4:0.2:0.4	80.43	0.78	0.89
0.5:0.2:0.3	68.48	0.68	0.81
0.3:0.2:0.5	64.13	0.64	0.81

## Discussion

This study demonstrates that multiteacher KD is an effective strategy for improving automated DPR-based radiographic scoring in a data-limited multiclass setting.^35^ The student model trained in the original DPR under KD supervision from 3 complementary teacher perspectives achieved 79.35% accuracy and an AUC of 0.88, substantially outperforming all 3 teachers despite being architecturally identical to each of them. It is acknowledged that the use of a uniform ResNet18 architecture across all models means that this study focuses on knowledge fusion across complementary preprocessing representations rather than model compression, which was a deliberate design choice to isolate the contribution of multiteacher distillation from architectural differences.

The 3 preprocessing strategies captured meaningfully different representations of the same DPR. The jaw-ROI crop (teacher 1) reduced background noise and improved focus on the anterior maxillary region, yielding the highest individual teacher accuracy (63.04%). The sharpened crop (teacher 2), while intuitively appealing for edge delineation, proved detrimental to score 3 classification, likely because sharpening amplified noise artefacts in low-contrast panoramic regions and introduced a distributional mismatch for that class. Teacher 3, trained on the original unmodified DPR, retained global contextual cues, including arch symmetry and bilateral structural references that aided minority class recognition, resulting in the highest AUC among teachers (0.80). Ablation experiments confirmed that the selected KD hyperparameters (*T* = 2.0, α = 0.7) were near-optimal. Lower temperatures reduced soft-label richness, while higher KD loss weights suppressed the cross-entropy signal, both of which degraded student performance. Analysis of individual teacher contributions further confirmed that all 3 teachers provided complementary supervisory information, with teacher 3 proving the most critical contributor. Teacher weighting analysis further revealed that reducing the contribution of teacher 2 while maintaining equal weights for teacher 1 and teacher 3 (0.4:0.2:0.4) improved accuracy to 80.43%, whereas strongly upweighting any single teacher markedly degraded performance, underscoring the importance of balanced complementary supervision between the jaw ROI crop and original DPR perspectives. The student, simultaneously learning from all 3 soft-label perspectives, synthesised complementary information unavailable to any single teacher, likely resulting in the substantial performance gain observed. The broader applicability of deep learning to dental radiographic assessment is further supported by recent work. Jiang et al^36^ demonstrated reliable automated detection of second-molar lesions related to impacted third molars on panoramic radiographs, while Ovuz et al^37^ reported accurate CNN-based identification of accessory mental foramina from cone-beam computed tomography, collectively reinforcing the translational relevance of artificial intelligence–assisted radiographic assessment across diverse dental anatomical structures and imaging modalities.

The 3 teacher networks exhibited rapid divergence between training and validation accuracy following backbone unfreezing at epoch 6, consistent with the known susceptibility of full fine-tuning to overfitting in small datasets. Despite this, the student network demonstrated a considerably more stable optimisation trajectory and a markedly reduced train-to-test gap, suggesting that KD provided implicit regularisation beyond what the freeze schedule and early stopping alone achieved in the teacher models.

A clinically important advantage of the proposed framework is that the student model operates exclusively on original, unmodified full DPR images during inference, eliminating the need for any preprocessing such as jaw-region cropping or sharpness enhancement. Despite receiving no cropped or enhanced input, the student implicitly learns to attend to the diagnostically relevant anterior maxillary region, as evidenced by the Grad-CAM visualisations in Figure 7, which show focused activation over the impacted canine and adjacent incisor roots. This is practically significant for clinical deployment, where preprocessing pipelines introduce additional computational steps undesirable in resource-constrained environments. Furthermore, the student network is computationally more efficient and demonstrates superior performance efficiency compared with the ensemble of multiple models. While ensembling requires the simultaneous availability of all input branches along with their corresponding input images, the proposed student KD network requires only a single unprocessed, unmodified full DPR for inference. Unlike most existing KD studies in medical imaging that focus on parameter reduction, the present approach prioritises clinical applicability, where the student accepts the simplest possible input, the original DPR, without any preprocessing burden, while substantially outperforming each individual teacher.

Score 3 remained the most challenging class across all models, with low recall despite relatively high precision, reflecting a conservative decision boundary. This class is both rare and potentially heterogeneous in its DPR appearance. The student’s Cohen’s κ of 0.58 represents moderate to substantial agreement and constitutes a meaningful clinical benchmark, compared with teacher κ values of 0.20 to 0.32, which would be considered insufficient for clinical use. Model calibration assessed via ECE further supported the student’s reliability, with the student achieving the lowest ECE (0.018) compared with all individual teachers and the teacher ensemble, indicating more consistent probabilistic predictions suitable for clinical decision support.

The limitations of this study include single-institution DPR data, a modest test set (n = 92), and very limited support for score 2 (n = 4). The small dataset size, despite augmentation to 300 images per class, remains the primary constraint on generalisation and should be addressed in future validation work. In particular, score 2 comprised only 4 test samples, rendering its perfect recall (1.00) and F-score (0.89) statistically unreliable, and these metrics should be interpreted with caution until validated on a larger score 2 sample. External validation across multi-institution DPR datasets remains an important future direction before clinical deployment of this system can be considered. While the proposed approach demonstrates strong performance, the differential teacher weighting scheme (0.4:0.2:0.4) explored in this study used fixed, manually defined weights; learnable or attention-based teacher weighting schemes that adapt dynamically during training are acknowledged as a promising direction for future work.

## Conclusion

This study evaluated multiteacher KD as a strategy for automated horizontal position scoring of impacted maxillary canines on panoramic radiographs. Three ResNet18 teacher models were independently trained on complementary DPR preprocessing variants: jaw ROI crops, sharpness-enhanced crops, and original full DPR, and their collective knowledge was distilled into a single student ResNet18 network. The student substantially outperformed every individual teacher in all metrics, achieving 79.35% accuracy, an AUC of 0.88, and Cohen’s κ of 0.58, supporting multiteacher KD as a practical and effective strategy for robust automated radiographic classification under limited annotated data conditions.

Future work should focus on prospective external validation across multi-institution DPR datasets to assess generalisability beyond the single-center setting evaluated here. Attention-based or learnable teacher weighting schemes should be explored as alternatives to the fixed differential weighting (0.4:0.2:0.4) employed in this study, as adaptive weighting may better capture class-level complementarity across teachers and further improve minority class discrimination beyond what fixed weighting achieves. The impact of larger or more capable backbone architectures, such as ResNet50 or EfficientNet,^38^ serving as teachers while the student remains lightweight, demands investigation, as richer soft-label supervision may further improve student generalisation. Additionally, expanding the annotated dataset, particularly for minority classes such as score 2 and score 3, remains a priority before the clinical deployment of such a system can be considered.

## Author contributions

R.C.H.: Conceptualisation, investigation, data curation, software, analysis and interpretation, writing—original draft preparation, writing—review and editing. V.M.: Conceptualisation, investigation, supervision, writing—review and editing. S.V.: Conceptualisation, supervision, writing—review and editing. V.P.: Conceptualisation, investigation, data curation, verification, writing—review and editing. A.S.: Review and editing.

## Ethics statement

Ethics approval was granted by the Institutional Ethics Committee (IEC) of the Manipal College of Dental Sciences, Manipal Academy of Higher Education, Manipal, India, under approval number 189/2023.

## Data availability

The data analysed during this study are not publicly available due to ethical and privacy restrictions but may be obtained from the corresponding author upon reasonable request. The source code for the ResNet18 framework is openly available on GitHub.^39^

## Conflict of interest

The authors declare that they have no known competing financial interests or personal relationships that could have appeared to influence the work reported in this paper.

## Appendix Supplementary materials

Image, application 1
