# Supplementary material for: Multiteacher Knowledge Distillation for Canine Scoring Using Dental Panoramic Radiographs to Support Primary Care
Source: Int Dent J. 2026 Jul 3;76(5):109711. doi: 10.1016/j.identj.2026.109711 (PMC13355671; doi:10.1016/j.identj.2026.109711)
Supplement: Supplementary file 1 [file mmc1.pdf]

Appendix 1. Checklist for AI in medical imaging (CLAIM) guidelines: 2024 Update.

Section / Topic	Item #	Description	Section*
**TITLE / ABSTRACT**			
	**1**	Identification as a study of AI methodology, specifying the category of technology used (e.g., deep learning)	Title
**ABSTRACT**			
	**2**	Summary of study design, methods, results, and conclusions	Abstract
**INTRODUCTION**			
	**3**	Scientific and/or clinical background, including the intended use and role of the AI approach	Introduction
	**4**	Study aims, objectives, and hypotheses	Materials and Methods: Study design
**METHODS**			
Study Design	**5**	Prospective or retrospective study	Materials and Methods: Study design
	**6**	Study goal	Materials and Methods: Study design
Data	**7**	Data sources	Materials and Methods: Study design
	**8**	Inclusion and exclusion criteria	Materials and Methods: Inclusion and exclusion criteria
	**9**	Data pre-processing	Materials and Methods: Dataset splitting and augmentation
	**10**	Selection of data subsets	Materials and Methods: Study design
	**11**	De-identification methods	Materials and Methods: Study design
	**12**	How missing data were handled	NA
	**13**	Image acquisition protocol	Materials and Methods: Study design
Reference Standard	**14**	Definition of method(s) used to obtain reference standard	Materials and Methods: Study design; Table 1,

	**15**	Rationale for choosing the reference standard	Introduction
	**16**	Source of reference standard annotations	Materials and Methods: Study design;
	**17**	Annotation of test set	Materials and Methods: Study design;
	**18**	Measures of inter- and intra-rater variability of features described by the annotators	Materials and Methods: Study design;
Data Partitions	**19**	How data were assigned to partitions	Materials and Methods: Dataset splitting and augmentation
	**20**	Level at which partitions are disjoint	Materials and Methods: Dataset splitting and augmentation
Testing Data	**21**	Intended sample size	Materials and Methods
Model	**22**	Detailed description of model	Materials and Methods: Model Architecture
	**23**	Software libraries, frameworks, and packages	Materials and Methods: Model Architecture
	**24**	Initialization of model parameters	Materials and Methods: Model Architecture
Training	**25**	Details of training approach	Materials and Methods: Training strategy
	**26**	Method of selecting the final model	Materials and Methods: Training strategy; Table 2
	**27**	Ensembling techniques	Materials and Methods: Training strategy
Evaluation	**28**	Metrics of model performance	Materials and Methods: Evaluation Metrics
	**29**	Statistical measures of significance and uncertainty	Results: Confidence Intervals; Table 6
	**30**	Robustness or sensitivity analysis	Results: Ablation Studies
	**31**	Methods for explainability or interpretability	Results: Student model performance; Figure 7
	**32**	Evaluation on internal data	Results
	**33**	Testing on external data	NC

	**34**	Clinical trial registration	NA
**RESULTS**			
Data	**35**	Numbers of patients or examinations included and excluded	Materials and Methods: Study design; Materials and Methods: Inclusion and exclusion criteria
	**36**	Demographic and clinical characteristics of cases in each partition	Materials and Methods
Model performance	**37**	Performance metrics and measures of statistical uncertainty	Results: Tables 3-4
	**38**	Estimates of diagnostic performance and their precision	Results: Confidence Intervals; Table 6
	**39**	Failure analysis of incorrect results	Results: Student model performance; Figure 6
**DISCUSSION**			
	**40**	Study limitations	Discussion
	**41**	Implications for practice, including intended use and/or clinical role	Discussion
**OTHER INFORMATION**			
	**42**	Provide a reference to the full study protocol or to additional technical details	NA
	**43**	Statement about the availability of software, trained model, and/or data	Data availability
	**44**	Sources of funding and other support; role of funders	Acknowledgment

* Indicate the page number and section title where each checklist item is addressed in the manuscript.

Here, NA denotes not applicable, and NC indicates that the activity was not carried out due to the unavailability of publicly accessible data.
